# Factors Influencing Antibody Response to SARS-CoV-2 Vaccination

**DOI:** 10.3390/vaccines11020451

**Published:** 2023-02-15

**Authors:** Cathrin Kodde, Sascha Tafelski, Efthimia Balamitsa, Irit Nachtigall, Marzia Bonsignore

**Affiliations:** 1 Department of Respiratory Diseases “Heckeshorn”, Helios Hospital Emil-von-Behring, 14165 Berlin, Germany; 2 Department of Anaesthesiology and Operative Intensive Care Medicine, Charité—Universitätsmedizin Berlin, 13353 Berlin, Germany; 3 Center for Hygiene, Evangelische Kliniken Gelsenkirchen, 45879 Gelsenkirchen, Germany; 4 Division of Infectious Diseases and Infection Prevention, Helios Hospital Emil-von-Behring, 14165 Berlin, Germany; 5 Institute of Hygiene and Environmental Medicine, Charité—Universitätsmedizin Berlin, 12203 Berlin, Germany; 6 Department of Infectious Diseases and Prevention, Helios Hospitals Duisburg, 47166 Duisburg, Germany; 7 Center for Clinical and Translational Research, Helios Universitätsklinikum Wuppertal, University of Witten/Herdecke, 42283 Wuppertal, Germany

**Keywords:** SARS-CoV-2, vaccinations, healthcare workers, public health, COVID-19

## Abstract

Vaccination plays a key role in tackling the ongoing SARS-CoV-2 pandemic but data regarding the individual’s protective antibody level are still pending. Our aim is to identify factors that influence antibody response following vaccination in healthcare workers. This single-center study was conducted at Evangelische Kliniken Gelsenkirchen, Germany. Healthcare workers were invited to answer a questionnaire about their vaccinations and adverse reactions. Subsequently, the level of anti-receptor binding domain (RBD) IgG antibody against SARS-CoV-2′s spike protein through blood samples was measured. For statistics, we used a defined correlation of protection (CoP) and examined risk factors associated with being below the given CoP. A total of 645 employees were included and most were female (n = 481, 77.2%). A total of 94.2% participants had received two doses of vaccines (n = 587) and 12.4% (n = 720) had been infected at least once. Most common prime-boost regimen was BNT162b2 + BNT162b2 (57.9%, n = 361). Age (*p* < 0.001), days since vaccination (*p* = 0.007), and the homologous vaccination regimen with ChAdOx + ChAdOx (*p* = 0.004) were risk factors for the antibody level being below the CoP, whereas any previous COVID-19 infection (*p* < 0.001), the number of vaccines (*p* = 0.016), and physical complaints after vaccination (*p* = 0.01) were associated with an antibody level above the CoP. Thus, age, vaccination regimen, days since vaccination, and previous infection influence the antibody level. These risk factors should be considered for booster and vaccinations guidelines.

## 1. Introduction

At the beginning of the pandemic and before vaccination programs were implemented, SARS-CoV-2 was able to hit an immunological naïve population, resulting in a fast spread around the globe with severe outcomes.

Since late 2020, vaccinations against the novel SARS-CoV-2 have been available and early studies showed an initial sufficient vaccination efficacy [1]. However, new variants such as the Delta variant (B.1.617.2) and subsequent variants became able to escape immune recognition and led to increased reports of vaccine breakthroughs [2,3]. 

To measure vaccine efficacy through the amount of antibodies, there are different quantitative assays that are used in the clinical setting. Some assays measure the number of neutralizing antibodies in IU/mL. Other assays calculate the ligand binding antibody units against SARS-CoV-2 spike protein or receptor binding domain (RBD), either as arbitrary units (AU/mL) or, after adjustment to the WHO standard, as binding antibody units (BAU/mL) [4]. 

However, the exact level of vaccine-induced antibody response that prevents infection (correlate of protection, CoP) is still pending. A hurdle in the determination of a commonly used CoP lies within the non-standardized assays globally used to detect serum antibody levels. Additionally, the immune defense against SARS-CoV-2 takes place through a complex interplay of cellular and humoral factors. Besides antibodies, the cellular immune response by SARS-CoV-2-specific T-lymphocytes plays an important role [5,6], which makes it difficult to predict the immune response based on a defined threshold alone. Measurement of antibodies is widely used and accepted, as the cellular immune responses are technically difficult to measure.

Besides a definitive CoP in order to classify vaccine efficacy, it is important to know what potential factors may influence the antibody level.

It is known that not only the different types of vaccines (e.g., inactivated, live-attenuated, toxoid) and time since vaccination can alter the immune response, but also intrinsic host factors such as comorbidities (e.g., obesity, cancer, cardiovascular, and autoimmune or chronic diseases) influence the immune response to vaccination [7,8,9]. Age is an important factor, as individuals have a lower vaccine response at the extremes of ages of life. Neonates have a less strong antibody production; the elderly, for example, have a more rapid antibody waning and a decline in antibody response to vaccinations [7,9]. Gender can also influence the antibody response; females are known to build a stronger and longer-lasting vaccine response than males due to genetic and hormonal differences [7,10,11]. At the same time, females have been shown to report more adverse effects following vaccination than males [7,12,13]. These factors are known to alter the antibody level after known vaccinations, but it is not fully known if it also applies for the COVID-19 vaccines. Prior studies examined reactogenicity and immunogenicity of vaccines among healthcare workers but focused on either side effects or serological antibody response [2,14]. 

Thus, our aim is to examine the antibody titer in vaccinated healthcare workers with possible associations to age, gender, time since vaccination, adverse reactions, and type of vaccine.

## 2. Materials and Methods

### 2.1. Patient Selection and Data Collection

On 22 September 2021, all employees (approximately 1400 persons) of the Evangelische Kliniken in Gelsenkirchen, Germany with a personal email address received an invitation for a free assessment of their antibody titer through a blood sample. To reach employees with no personal email address, the invitation was posted on the hospital’s intranet top news page; additionally, it was printed and displayed in prominent areas of the hospital. The letter included a questionnaire in which participants were asked to complete if were participating. Participation was restricted to employees of the Evangelische Kliniken. Employees who, according to their questionnaire, had no history of vaccination still received antibody testing, but were not included in the study cohort.

For antibody analysis, we used the fully automated access SARS-CoV-2-IgG test by BeckmanCoulter^©^. This semi-quantitative assay measures IgG antibodies in blood samples directed against the receptor-binding domain of the S-protein of SARS-CoV-2, giving numerical result in arbitrary units (AU) from 2.00 to 450 AU/mL [15]. One major study calculated a CoP against COVID-19 infection of 54 IU/mL [5]. The study examined data from clinical COVID-19 vaccination trials and correlated neutralizing antibody levels with vaccine efficacy [5,16]. This threshold will be used as a reference in our study. Studies showed that AU can be directly compared to IU, because they have a strong, significant correlation. IU measures neutralizing antibodies such as IgG, IgM, and IgA, whereas Anti-RBD IgG antibody is measured in AU. In those studies both neutralizing antibodies and Anti-RBD IgG antibody levels were quantified and a strong correlation was found between these two. Thus, anti-RBD IgG antibody levels are used to assess a level of protection [17,18,19].

Written informed consent was received by all participants. Participation to the survey was voluntary and anonymous. The included questionnaire was developed by infectious diseases experts and the translations of the question can be found in Table 1. All information derived from the questionnaire and was self-reported.

### 2.2. Ethical Consent

The study was approved by the Ethic Committee of the General Medical Council (Ärztekammer) of Westfalen-Lippe (2021-573-f-S) and registered in the German Clinical Trials Register (DRKS 00027266).

### 2.3. Statistical Analysis

Data were analysed using IBM Statistics^®^ (Vs 28.0, IBM corporation, 2021). For descriptive analysis, we presented data with numbers and percentage in nominal data and used mean and 95% confidence interval in discrete data. Median and interquartile range was used in ordinal data and variables with non-normal distributions. For analysis of statistical significance, we used Fisher’s exact test, t-test, or the Mann–Whitney tests according to scale level and data distribution, respectively. In analysis with more than two groups, the Kruskal–Wallis test was used when pre-assumptions for ANOVA were fulfilled. Furthermore, we applied multivariate logistic regression analysis as a prediction model for a clinically meaningful antibody thresholds of 54 IU/mL [5]. Following transformation of measured antibody titers into a binary-dependent variable, covariables were defined based on scientific evidence. Therefore, age, gender, type of immunisation, duration in days since last known contact to COVID antigen or incidence of COVID infection, number of vaccinations, and physiologic response to vaccination entered the regression model. For regression analysis, we reported odds ratio and 95% confidence interval for each variable and corresponding significance level. For all statistical analysis, we used a *p* value of ≤0.05 as significance level. To further explore findings of the multivariate regression analysis, we plotted resulting predicted probabilities of the model against age groups and type of vaccination. Therefore, data were visualized with error bars with mean and 95% confidence intervals.

Due to the observational design of the study, alpha cumulation was not addressed as results were seen as an explorative analysis.

## 3. Results

### 3.1. Descriptive and Inferential Statistics Results

**Figure 1:**
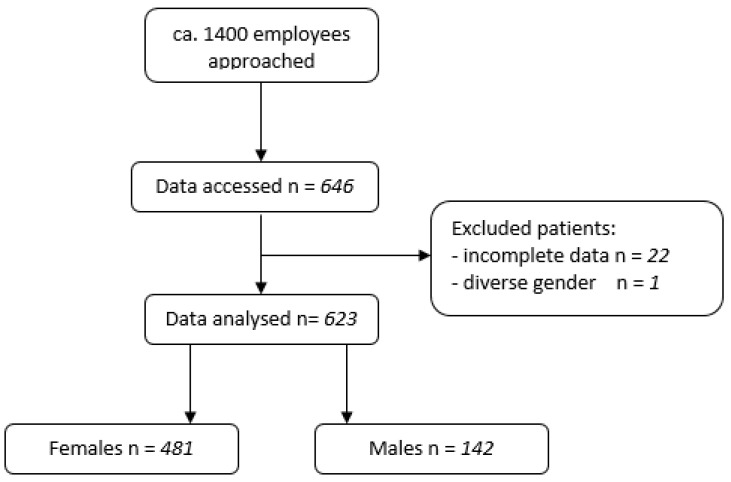
Flowchart of included patients.

In total, 646 employees consented to participate in this study, of which 22 employees gave incomplete data or incoherent information about previous vaccinations and were excluded from the study (see Figure 1).

Out of these, 481 (77.21%) were female. One participant was diverse. With regard to this single diverse participant, no separate analysis was carried out. Baseline characteristics are described in Table 2.

The most common prime-boost regimen was BNT162b2 + BNT162b2 (57.9%, n = 361), followed by the heterologous combination ChAdOx + BNT162b2 (20.2%, n = 126) and ChAdOx + ChAdOx (13%, n = 81). By the time of examination, most of the employees had received two doses of vaccines (94.2%, n = 587), 12.4% (n = 77) had been infected with SARS-CoV-2 at least once.

The vast majority of participants reported physical complaints after vaccination (89.1%, n = 555). The most frequently stated complaint was pain at the injection site (84.8%, 528/623), followed by tiredness (73.5%, 458/623), and muscle or joint pain (62.3%, 388/623). Severe adverse events including neurological disorders (Guillain–Barré syndrome or facial nerve paralysis) occurred in one case (0.1%, 1/623) and cardiac complications (myocarditis/pericarditis or arrhythmias) were self-reported in 8 cases (1.3%, 8/623). No gender-specific differences in reactogenicity frequency was observed. The participants could rank their physical complaints according to an ordinal scale from 1 = mild, 2 = moderate, 3 = severe for each time they got vaccinated. We saw that females reported statistically significant stronger physical complaints (female mean 2.01–95% confidence interval (CI) 1.88–2.15; male mean 1.62; 95% CI 1.39–1.86; *p* = 0.008).

**Figure 2:**
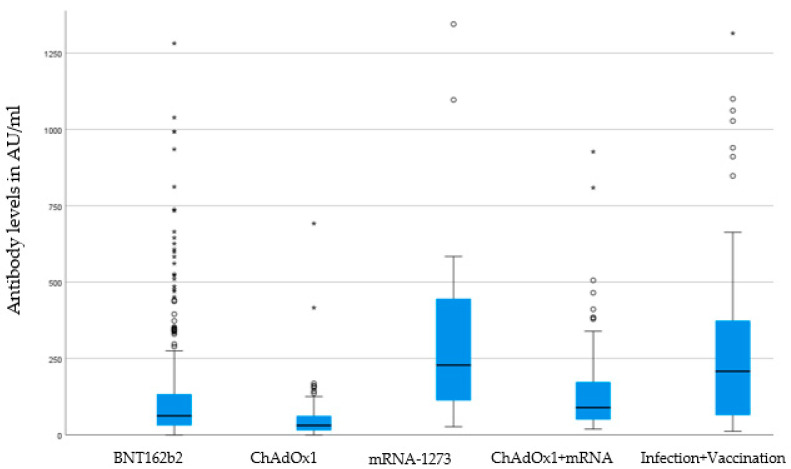
Boxplot of antibody levels according to vaccines. Boxes show median and interquartile range (IQR) between 25% quartile (Q1) and 75% quartile (Q3), whiskers (lower whisker Q1-1.5*IQR and upper whisker Q3 + 1.5*IQR), outliers (asterisks), and extreme values (circles). Analysis of statistical significance *p* < 0.001 (Kruskal-Wallis-test). *BNT162b2* (Pfizer–BioNTech), *mRNA*-1273 (Moderna), and *ChAdOx1* (AstraZeneca).

The homologous schedule with mrNA-1273 (mean 383,4 AU/mL—95% CI 192.58–574.2) and the heterologous schedule ChAdOx1 + mRNA vaccine (mean 136,12 AU/mL—95% CI 109.32–162.92) showed the highest antibody level, followed by BNT162b2 (mean 135 AU/mL—95% CI 112.78–157.38). The lowest antibody titer was recorded for the homologous vaccine scheme with ChAdOx1 (mean 57.58 AU/mL—95% CI 36.40–78.76) (see Figure 2 “Boxplot of antibody level”). We found no gender differences in the height of antibody titer (male: mean 184 AU/mL—95% CI 134–233; female: 155 AU/mL—95% CI 133–177; *p* = 0.944).

### 3.2. Multivariate Logistic Regression Results

To identify possible factors influencing the antibody titer we defined the null hypothesis as the above-mentioned antibody neutralizing level of 54 IU/mL. If it was below, we rejected the null hypothesis. By using a multivariate analysis, we identified different parameters that are associated with a CoP < 54 IU/mL (see Table 3 “Multivariate analysis of influencing factors”).

**Figure 3:**
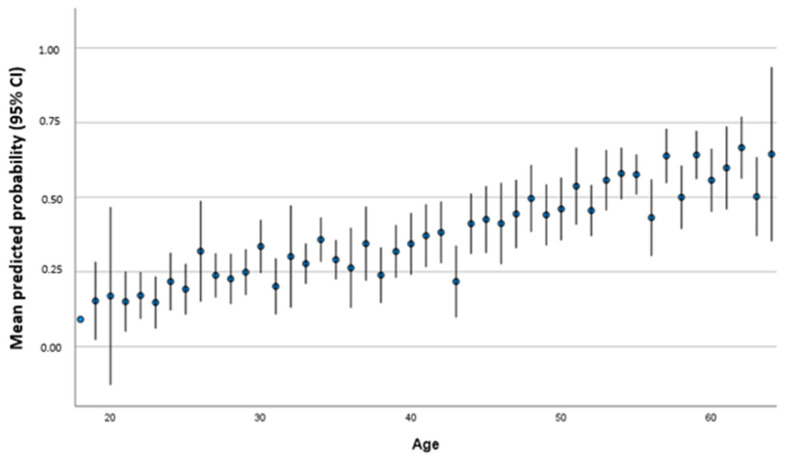
Mean predicted probability (95% CI) to achieve a threshold below 54 IU/mL according to age in years.

For each year of life, the risk increased that the titer was not above the threshold (*p* < 0.001; OR 1.047; 95% CI 1.031–1.063). In Figure 3, we differentiated age groups according to predicted probability for persons achieve a threshold below 54 IU/mL of antibody titer based on the multivariate regression model that shows an increase in probability with increasing age in concordance with Table 3.

**Figure 4:**
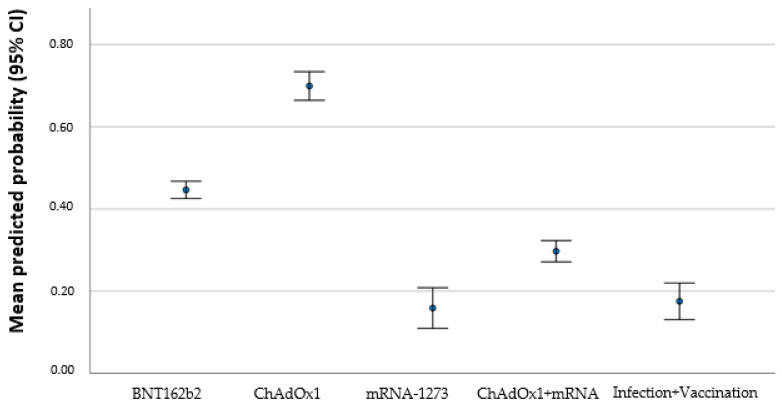
Mean predicted probability (95% CI) to achieve a threshold below 54 IU/mL according to different vaccines. *BNT162b2* (Pfizer–BioNTech), *mRNA*-1273 (Moderna), and *ChAdOx1* (AstraZeneca).

The homologous scheme with ChAdOx yield a significant risk to result in a titer under the given threshold (*p* = 0.004; OR 15.159; 95% CI 2.34–98.22). Individuals that received more than one vaccination were more likely to have antibody level above the threshold: each additional vaccination increases the possibility to be above the threshold (*p* = 0.016 OR 0.150; 95% CI 0.032–0.700). Additionally, it was more likely that the individuals’ antibody titer was above the given threshold when somebody was infected before or after vaccination. Longer time since vaccination was associated with an antibody level below threshold (*p* = 0.007 OR 1.005 95% CI 1.001–1.009). Figure 4 shows the predicted probability for persons to reach a threshold below 54 IU/mL of antibody titer based on the multivariate regression model that shows an increase in probability with regard to different vaccines schedules.

A high reactogenicity was associated with an antibody level above the threshold (*p* = 0.010 OR 0.842 95% CI 0.739–0.960).

To report the area under the curve (AUC) and c-statistics for the multivariate logistic regression we used an analysis model fit using a Hosmer–Lemeshow test for the model that demonstrated sufficient model quality. To further backup this analysis, we performed requested c- statistics for reported model. Our results showed a suitable c-statistics with an AUC of 0.780 [95% CI 0.743–0.817] (see Supplementary Materials, Figure S1).

## 4. Discussion

### 4.1. Summary and Contributions

Higher age, more days since last antigen contact, and a homologous vaccination with ChAdOx were associated with higher odds for an antibody level below threshold. Numerous vaccinations and an additional infection led to lower odds. Although women reported a higher severity of experienced reactogenicity, gender did not influence the odds for an antibody titer below threshold.

Our study reports a negative correlation between age and antibody titer; with each year of life, it was more likely that individuals did not reach the given threshold. It is widely known that elderly people have a lower antibody response to different vaccinations such as hepatitis B, seasonal influenza, tetanus, and pneumococcal vaccine [7,20,21]. In contrast to young individuals, the elderly have a rapid waning rate of neutralizing antibodies that makes them more susceptible for vaccine-preventable diseases. This age-related decrease in neutralization level is due to the decline of innate and adaptive immunity [21].

We saw a positive correlation of vaccine-induced symptoms with antibody levels. To this date, no clear picture is drawn whether more side effects means higher antibody levels or not [22,23,24]. However, more recent studies saw a positive correlation between reactogenicity and immunogenicity [24,25]. The underlying physiological mechanism is not well studied yet. Our study supports the notion that an intensive physical response promotes a stronger antibody response.

Our study demonstrated that females were more likely to report stronger side effects after vaccination than males consistent with other studies [12,22]. Females reported higher levels of reactogenicity with more moderate to severe physical complaints after vaccination. The height of reactogenicity has been associated with the height of immunogenicity for other vaccines [7,10]. However, we found no correlation between gender and the risk for an antibody titer below threshold.

Generally, it is known that females tend to have higher antibody response than males. These findings apply for the majority of vaccines including hepatitis A and B, seasonal influenza, Haemophilus influenza A, yellow fever, and measles [7,10]. Males on the other hand have a higher antibody response to tetanus and diphtheria vaccines [7,26,27]. The females’ innate and adaptive immune system responds faster to vaccinations, not only contributing to stronger immunogenicity but also higher reactogenicity because of hormonal, behavioral, and genetic factors [12,13,28,29]. However, recent studies on gender-specific differences of SARS-CoV-2 vaccine-induced immunity yield conflicting results. In accordance with our study, no gender-based differences in antibody level were also found in other studies [22,25,28,30].

We report that the different vaccination regimen had a significant influence on the immune response. The homologous vaccine regimen with ChAdOx resulted in a higher risk of being below the given threshold. This finding is in accordance with recent studies where this vaccine regimen led to a weaker immune response, whereas BNT162b2/BNT162b2 vaccines elicit a stronger antibody response [31,32]. Studies also showed that heterologous vaccines regimen mostly ChAdOx/BNT162b2 tend to have a stronger immunogenicity than homologous BNT162b2 regimen because of the additional effect of each vaccine on the humoral immune system response [31,33]. However, our data did not confirm these results. This finding may not be representative as the vast majority of our study population has received the homologous BNT162b2/ BNT162b2 regimen, as this was the first authorized vaccine and primarily used in the clinical setting.

Comparable antibody levels where seen in our study between BNT162b2/BNT162b2 regimen and infected participants who received one additional dose of any vaccination. Immunological studies assume that a natural infection elicits and triggers a broader humoral immune response [34]. Thus, vaccinations prior or post-infection act as a booster in individuals [34,35].

With regard to our results, we recommend that people should get at least two vaccinations, as every additional contact to the virus through either vaccination or through natural infection increases the immune response. Elderly people should know that they may have a lower antibody response and thus frequent boosters may be required.

In general, a booster vaccination is important to maintain a sufficient antibody level and thus protection from infection. In our study, we saw that each administered vaccinations reduced the risk of being below the threshold. Time since vaccination is a widely known risk factor for waning antibodies levels [30,31], which was also demonstrated in our study.

### 4.2. Strengths and Limitations

The strength of our study is the comparable high number of participants and the use of real-world data. Our study has some limitations that may have biased the results.

One limitation is the given threshold in IU/mL measuring neutralizing antibodies including IgG, IgM, and IgA, whereas our used assay detects specifically Anti-RBD IgG antibody of the S-protein of SARS-CoV-2 (AU/mL). However, studies showed a strong correlation between both levels. Anti-RBD IgG antibody is clinically used to assess a level of protection as it comprise for about 90% of neutralizing activity [18,19]. Our cohort consisted only of workers from the healthcare sector, naturally including more females than males in the working age range. Thus, male gender and younger or elderly individuals are underrepresented in our study. We included a sample with imbalanced sample sizes that was not modifiable in this trial. Despite observed differences between groups were evaluated with statistical tests, imbalanced sample sizes could result in reduced power and therefore results should be assessed in further trials. Participants with intense side effects may have led to an over reporting as they wanted to share their experiences through the questionnaire.

We also relied on self-reporting regarding the side effects, number of vaccinations, and infections. We have not performed an anti-spike/anti-nucleocapsid antibody test to identify all previously infected participants, which may have biased the outcome of antibody level. Furthermore, potential interactions of variables were not addressable in this analysis due to limited sample size; however, sexes may differ in more dimensions such as pain-, infection-, or vaccination response [36,37,38].

### 4.3. Future Work

Distinct studies should be carried out to see if risk factors for a lower or higher antibody response are proven. Further studies are important to establish booster guidelines with regard to individual risk factors for a lower antibody response and thus the need for a booster vaccination. Some influencing factors such as age and previous infection are already known. Additionally, our study supports the assumption that a stronger reactogenicity results in a higher antibody response, especially in females. Thus, gender specific research regarding antibody level and side effects should be run. It is also important to examine if the same risk factor applies for mRNA vaccinations as well as traditional vaccinations (e.g., live, inactivated, or conjugate vaccines).

## 5. Conclusions

Our study identifies different factors influencing antibody response to SARS-CoV-2 vaccination such as age, vaccination regimen, days since vaccination, and previous infection. The findings are important with regard to vaccination and booster guidelines for COVID-19 as we were able to identify risk factors for a lower antibody response.

## Figures and Tables

**Table 1:** Questionnaire.

Gender	☐ Woman
	☐ Man
	☐ Diverse
	
Immunosuppressive medication	☐ yes (name of drug)
	☐ no
	
Vaccination	☐ first
	☐ second
	☐ third
	
Vaccine	☐ BNT162b
	☐ ChAdOx 2
	☐ mRNA-1273
	
Reactions (multiple answers possible range from low, medium, strong to very strong)	☐ none
	☐ pain at the injection site
	☐ pain/swelling at injection arm
	☐ fever
	☐ tiredness
	☐ headache
	☐ chills
	☐ muscle pain
	☐ joint pain
	☐ nausea/vomiting
	☐ diarrhoea
	☐ anaphylactic reaction
	☐ myocarditis/pericarditis
	☐ facial nerve paralysis
	☐ cardiac arrhythmia
	☐ Guillan-Barré-Syndrome
	☐ thrombosis with thrombocytopenia
	☐ sinus venous thrombosis, arterial thrombosis
	☐ capillary leak syndrome
	
Recovered from COVID-19	☐ yes
	☐ no

**Table 2:** Baseline characteristics.

Parameters	Female	Male	*p*-Value	Odds Ratio (95% Confidence Interval)
Participants (total =)	481	142		
Age median (IQR)	44.38 22 (33–55)	43.6 22 (33–55)		
Recovered after COVID-19 N (%)	62 (12.9)	15 (10.6)		1.148 0.650–2.027
1st vaccine (total = 623 patients)				
BNT162b2 N (%)	305 (63.4)	79 (55.6)	0.253	
ChAdOx 2 N (%)	161 (33.5)	56 (39.4)		
mRNA-1273 N (%)	12 (2.5)	7 (4.9)		not available
Jcovden	2 (0.4%)	0		
without 1. vaccination N (%)	1 (0.2%)	0		
2nd vaccine (total = 621 patients)				
BNT162b2 N (%)	385 (80.4%)	100 (70.4%)	0.002	
ChAdOx 2 N (%)	53 (11.1)	32 (22.5)		
mRNA-1273 N (%)	11 (2.3)	6 (4.2)		not available
without 2. vaccination N (%)	30 (6.3%)	4 (2.8%)		
3rd vaccine (total = 527 patients)				
BNT162b2 N (%)	11 (2.7)	6 (4.8)	0.088	
ChAdOx 2 N (%)	0	1 (0.8)		
mRNA-1273 N (%)	0	0		not available
without 3. Vaccination N (%)	391 (97.3%)	118 (94.4)		
Vaccine combination. if >1 vaccination				
BNT162b2 + BNT162b2 N (%)	285 (59.3)	76 (53.5)	0.246	1.263 0.866–1.840
mRNA-1273 + mRNA-1273 N (%)	10 (2.1)	6 (4.2)	0.221	0.481 0.172–1.348
ChAdOx + ChAdOx N (%)	50 (10.4)	31 (21.8)	0.001	0.415 0.253–0.681
ChAdOx + BNT162b2 N (%)	102 (21.2)	24 (16.9)	0.286	1.323 0.810–2.161
ChAdOx + mRNA-1273 N (%)	1 (0.2)	0		
Reactions and complications after any vaccination (Multiple answers were possible) Based on 1227 vaccinations with information on reactions and gender				
None N (%)	46 (9.6)	22 (15.5)	0.065	0.577 0.334–0.997
Headache N (%)	285 (58)	63 (44.4)	0.002	1.823 1.250–2.660
Pain at injection site N (%)	417 (86.7)	111 (78.2)	0.017	1.820 1.129–2.933
Rush at injection site N (%)	116 (23.3)	39 (26.5)	0.442	0.841 0.552–1.281
Tiredness N (%)	362 (75.3)	96 (67.6)	0.083	1.458 0.969–2.192
Fever/Chills N (%)	236 (489.1)	66 (46.5)	0.633	1.109 0.762–1.614
Gastrointestinal complaints (diarrhea, Nausea, vomiting) N (%)	59 (12.3)	13 (9.2)	0.371	1.387 0.737–2.610
Neurological complications (Facialis+ Guillan) N (%)	0	1 (0.7)	0.228	not available
Thrombotic complications N (%)	0	0	-	
Cardiac complications N (%)	6 (1.2)	2 (1.4)	1	0.884 0.177–4.430
Muscle or joint pain N (%)	309 (62)	82 (55.8)	0.180	1.296 0.893–1.881
Allergic reaction N (%)	11 (2.3)	3 (2.1)	1	1.084 0.298–3.942
Antibody titer Mean	155 AU/mL	184 AU/mL	0.944	160.02 140.23–179.80

IQR—interquartile range, OR—odds ratio, *n*—number, AU/mL—arbitrary units per mL; *BNT162b2* (Pfizer–BioNTech), *mRNA*-1273 (Moderna), *ChAdOx1* (AstraZeneca), and *Jcovden* (Johnson&Johnson).

**Table 3:** Multivariate analysis of influencing factors being below the threshold of 54 IU/mL. *BNT162b2* (Pfizer–BioNTech), *mRNA*-1273 (Moderna), and *ChAdOx1* (AstraZeneca).

	*p*-Value	Odds Ratio (95% Confidence Interval)
Age	<0.001	1.047 1.031–1.063
gender (female versus male)	0.509	1.166 0.740–1.837
If BNT162b2 + BNT162b2	0.100	4.835 0.740–31.603
If mRNA-1273 + mRNA-1273	0.858	1.232 0.126–12.063
If ChAdOx + ChAdOx	0.004	15.159 2.340–98.222
If ChAdOx + BNT162b2	0.316	2.404 0.434–13.326
days since antigen contact (infection or vaccination)	0.007	1.005 1.001–1.009
any infection	<0.001	0.111 0.044–0.279
Number of vaccines	0.016	0.150 0.032–0.700
Physical complaint	0.010	0.842 0.739–0.960

## Data Availability

The data presented in this study are available on request from the corresponding author. The data are not publicly available due to strict regulations regarding privacy.

## Supplementary Materials

The following supporting information can be downloaded at: https://www.mdpi.com/article/10.3390/vaccines11020451/s1, Figure S1: Area under the curve (AUC) and c-statistics for the multivariate logistic regression that showed a suitable c-statistics with an AUC of 0.780 [95% CI 0.743-0.817].

## Abbreviations

IU	International Units
BAU	Binding Antibody Units
AU	Arbitrary Units
RBD	Receptor Binding Domain
CoP	Correlate of Protection
CI	Confidence Interval
AUC	Area Under the Curve
