# Supplementary material for: Factors Influencing Antibody Response to SARS-CoV-2 Vaccination
Source: Vaccines (Basel). 2023 Feb 15;11(2):451. doi: 10.3390/vaccines11020451 (PMC9967627; doi:10.3390/vaccines11020451)
Supplement: Supplementary file 1 [file vaccines-11-00451-s001.zip › vaccines-2137658-supplementary.pdf]

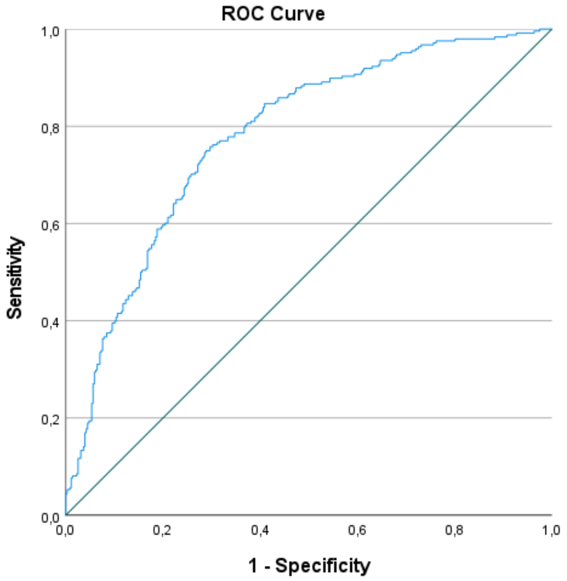

Figure S1. Area under the curve (AUC) and c-statistics for the multivariate logistic regression that showed a suitable c-statistics with an AUC of 0,780 [95% CI 0.743-0.817].
